# Supplementary material for: OpenMM 7: Rapid development of high performance algorithms for molecular dynamics
Source: PLoS Comput Biol. 2017 Jul 26;13(7):e1005659. doi: 10.1371/journal.pcbi.1005659 (PMC5549999; doi:10.1371/journal.pcbi.1005659)
Supplement: S1 Source Code — (ZIP) [file pcbi.1005659.s002.zip › OpenMM-7.0.1-Source/docs/api-c++/generated/OpenMM.CompoundIntegrator.html]

CompoundIntegrator — OpenMM @OPENMM\_MAJOR\_VERSION@.@OPENMM\_MINOR\_VERSION@ documentation


# CompoundIntegrator¶

*class* `OpenMM::``CompoundIntegrator`¶
:   This class allows you to use multiple integration algorithms within a single simulation, switching back and forth between them. To use it, create whatever other Integrators you need, then add all of them to a `CustomIntegrator`:

    ```
    CompoundIntegrator compoundIntegrator;
    compoundIntegrator.addIntegrator(new VerletIntegrator(0.001));
    compoundIntegrator.addIntegrator(new LangevinIntegrator(300.0, 1.0, 0.001));
    ```

    Next create a `Context`, specifying the `CompoundIntegrator` as the `Integrator` to use for the `Context`:

    ```
    Context context(system, compoundIntegrator);
    ```

    Finally, call `setCurrentIntegrator()` to set which `Integrator` is active. That one will be used for all calls to `step()` until the next time you change it.

    ```
    compoundIntegrator.setCurrentIntegrator(0);
    compoundIntegrator.step(1000); // Take 1000 steps of Verlet dynamics
    compoundIntegrator.setCurrentIntegrator(1);
    compoundIntegrator.step(1000); // Take 1000 steps of Langevin dynamics
    ```

    When switching between integrators, it is important to make sure they are compatible with each other, and that they will interpret the positions and velocities in the same way. Remember that leapfrog style integrators assume the positions and velocities are offset from each other by half a time step. When switching between a leapfrog and non-leapfrog integrator, you must first adjust the velocities to avoid introducing error. This is also true when switching between two leapfrog integrators that use different step sizes, since they will interpret the velocities as corresponding to different times.

    Methods

    |  |  |
    | --- | --- |
    | `CompoundIntegrator` | Create a `CompoundIntegrator`. |
    | `~CompoundIntegrator` |  |
    | `getNumIntegrators` | Get the number of Integrators that have been added to this `CompoundIntegrator`. |
    | `addIntegrator` | Add an `Integrator` to this `CompoundIntegrator`. |
    | `getIntegrator` | Get a reference to one of the Integrators that have been added to this `CompoundIntegrator`. |
    | `getIntegrator` | Get a const reference to one of the Integrators that have been added to this `CompoundIntegrator`. |
    | `getCurrentIntegrator` | Get the index of the current `Integrator`. |
    | `setCurrentIntegrator` | Set the current `Integrator`. |
    | `getStepSize` | Get the size of each time step, in picoseconds. |
    | `setStepSize` | Set the size of each time step, in picoseconds. |
    | `getConstraintTolerance` | Get the distance tolerance within which constraints are maintained, as a fraction of the constrained distance. |
    | `setConstraintTolerance` | Set the distance tolerance within which constraints are maintained, as a fraction of the constrained distance. |
    | `step` | Advance a simulation through time by taking a series of time steps. |

    `CompoundIntegrator`()¶
    :   Create a `CompoundIntegrator`.

    `~CompoundIntegrator`()¶

    int `getNumIntegrators`() *const*¶
    :   Get the number of Integrators that have been added to this `CompoundIntegrator`.

    int `addIntegrator`(Integrator \**integrator*)¶
    :   Add an `Integrator` to this `CompoundIntegrator`. The `Integrator` object should have been created on the heap with the “new” operator. The `CompoundIntegrator` takes over ownership of it, and deletes it when the `CompoundIntegrator` itself is deleted. All Integrators must be added before the `Context` is created.

        |  |  |
        | --- | --- |
        | Parameters: |  |

        - **integrator** – the `Integrator` to add

        |  |  |
        | --- | --- |
        | Returns: | the index of the `Integrator` that was added |

    Integrator &`getIntegrator`(int *index*)¶
    :   Get a reference to one of the Integrators that have been added to this `CompoundIntegrator`.

        |  |  |
        | --- | --- |
        | Parameters: |  |

        - **index** – the index of the `Integrator` to get

    *const* Integrator &`getIntegrator`(int *index*) *const*¶
    :   Get a const reference to one of the Integrators that have been added to this `CompoundIntegrator`.

        |  |  |
        | --- | --- |
        | Parameters: |  |

        - **index** – the index of the `Integrator` to get

    int `getCurrentIntegrator`() *const*¶
    :   Get the index of the current `Integrator`.

    void `setCurrentIntegrator`(int *index*)¶
    :   Set the current `Integrator`.

        |  |  |
        | --- | --- |
        | Parameters: |  |

        - **index** – the index of the `Integrator` to use

    double `getStepSize`() *const*¶
    :   Get the size of each time step, in picoseconds. This method calls `getStepSize()` on whichever `Integrator` has been set as current.

        |  |  |
        | --- | --- |
        | Returns: | the step size, measured in ps |

    void `setStepSize`(double *size*)¶
    :   Set the size of each time step, in picoseconds. This method calls `setStepSize()` on whichever `Integrator` has been set as current.

        |  |  |
        | --- | --- |
        | Parameters: |  |

        - **size** – the step size, measured in ps

    double `getConstraintTolerance`() *const*¶
    :   Get the distance tolerance within which constraints are maintained, as a fraction of the constrained distance. This method calls `getConstraintTolerance()` on whichever `Integrator` has been set as current.

    void `setConstraintTolerance`(double *tol*)¶
    :   Set the distance tolerance within which constraints are maintained, as a fraction of the constrained distance. This method calls `setConstraintTolerance()` on whichever `Integrator` has been set as current.

    void `step`(int *steps*)¶
    :   Advance a simulation through time by taking a series of time steps. This method calls `step()` on whichever `Integrator` has been set as current.

        |  |  |
        | --- | --- |
        | Parameters: |  |

        - **steps** – the number of time steps to take

High performance molecular simulation on GPUs


### Navigation

- Core Objects
- Forces
- Integrators
- Extras

©2015, Stanford University and the Authors.
|
Powered by Sphinx 1.3.1
& Alabaster 0.7.7
|
Page source
